# Effects of Facility Adaptation Promotion Program for Korean Older Adults in Nursing Home

**Published:** 2019-11

**Authors:** Sohyune R SOK

**Affiliations:** College of Nursing Science, Kyung Hee University, Seoul, Republic of Korea

**Keywords:** Aged, Nursing home, Adaptation, South Korea

## Abstract

**Background:**

The purpose of this study was to examine the effects of facility adaptation promotion program on self-esteem, depression, relationship, life satisfaction, and adaptation to facility of Korean older adults in nursing home.

**Methods:**

A quasi-experimental pretest-posttest control group design was employed. Study participants were a total of 73 older adults aged 65 yr and older (Experimental: n=36, Control: n=37) who were living at nursing home in Seoul, South Korea in 2016. They were recruited through convenient sampling. Measures were Self-esteem scale, Korean Geriatric Depression Scale, Relationship Change Scale, life satisfaction scale, and facility adaptation scale. Data were analyzed using the SPSS version 21.0 with descriptive statistics, the Chi-squared test, and independent t-test.

**Results:**

Facility adaptation promotion program increased self-esteem (t=19.067, *P*<0.001), relationship (*t*=24.533, *P*<0.001), life satisfaction (*t*=16.501, *P*<0.001), and adaptation to facility (*t*=24.328, *P*<0.001), and decreased depression (*t*=14.491, *P*<0.001) of Korean older adults in nursing home.

**Conclusion:**

Facility adaptation promotion program can be implied for improving self-esteem, relationship, life satisfaction, and adaptation to facility, and for decreasing depression of Korean older adults in nursing home.

## Introduction

South Korea has been experiencing the fastest aging in the world (1). The older adult population aged 65 years or older in the South Korea accounted for 13.7% of the total population in 2016 while it was 8.5% in the world (1). In 2026, the number of older adults in South Korea will reach about 20% to enter the super-aged society (1). The phenomenon of rapid population aging has caused not only individual health problems, but also various social issues, such as the increase in the burden on the family and society for caring for the elderly, and family members experiencing considerable physical, psychological, and social challenges in providing long-term care for the elderly (2, 3). Such a phenomenon will lead to an increase in the number of elderly who will be removed from their families and will spend their life in nursing facilities, senior care group homes, or other types of senior care facilities (2, 4). Despite the growing number of the elderly who enter into such facilities, there is a huge lack of various conditions that can accommodate them. As a result, there is an increasing importance of the adaptation of the elderly after having been admitted to these facilities.

Being admitted to a senior care facility is considered as a critical moment in an elderly person's life (5, 6). Furthermore, many elderly people experience a sense of loss, and they think that it would mark a drastic severance from the closest people in their life who were linked to them until now (2, 7). It would become more stressful for the elderly, who were admitted to the facility regardless of their willingness (8, 9), thereby causing more negative emotions, such as worthlessness or lethargy (10–12). The elderly's voluntary and involuntary movement of residence (relocation) allows them to experience many changes in their daily lifestyle pattern, social network, and support system that they have established up to now (11). This may cause an environmental change stress syndrome (Relocation stress syndrome; NANDA-I). In addition, the elderly who are admitted to a facility often show symptoms, such as anxiety, confusion, insomnia, diminished appetite, depression, loneliness, lethargy, or tearing (2, 8, 13), while in serious cases, they show a non-adaptive behavior, such as attempting or committing suicide (5, 9, 12, 14).

Most Korean and foreign studies have focused on qualitative research or fragmented factor analyses on the elderly's experience of facilities (6, 8, 11, 15). While there were some qualitative studies on facility adaptability and post-admittance experience, they mainly concluded their research by deriving and describing conceptual qualitative research methods. Furthermore, existing studies related to programs have not been adequately implemented, and most programs currently in place are the ones that simplified the leisure programs for adults in general (2, 4, 16, 17). Despite the continuous research on the elderly's adaptability to these facilities, the development and implementation of programs that can actually help the elderly with their adaptability in the facilities are still lacking. Therefore, it is necessary to conduct a research on developing and verifying the implementation effects of systematic and coordinated facility adaptation promotion program for the elderly, who are admitted to long-term care facilities, so as to promote their adaptation to new environments.

Therefore, the aims of this study were to examine effects of facility adaptation promotion program on self-esteem, depression, relationship, life satisfaction, and adaptation to facility of older adults in nursing home, South Korea.

## Materials and Methods

### Study design and Sample

A quasi-experimental pretest-posttest control group design was employed. Study sample included a total of 73 older adults who were living at nursing home in Seoul, South Korea. They were recruited through convenient sampling, and were randomly assigned to experimental group (n=36) and control group (n=37). The eligibility criteria included an age of 65 years or older, understanding the purpose of this study, consent to participate in the study, having no cognitive impairment (24 scores above by MMSE-K), and having complete verbal communication ability in Korean.

Sample size adequacy (n=26 in each group) using t test, G power 3.1 analysis software was estimated based on an alpha level=0.05, effect size=0.8, and power=0.8 (18). Therefore, the sample size in the study was adequate.

### Experimental Intervention

The experimental intervention in this study was a facility adaptation promotion program developed by Chang and Park^10^ based on the results from the elderly's program demand level survey, as well as the previous studies on the elderly's adaptation to the nursing facilities and intervention programs that used empowerment (19–23). A 10-week program has been developed, in which each 60-minute session consisted of group education and group discussion. The facility adaptation promotion program was conducted in the order of the verification of target objectives, experience sharing, information sharing, group discussions among members, and summarization. As the program started, the participants shared information whether they had achieved the objectives that they set for the last week, experiences on problem solving, and acquired knowledge and skills required for promoting their adaptability to the facilities through group education. Based on the information offered after the education, the members verified their own problems related to the main theme, and they actively participated in the group discussion, so as to discover solutions that are most suitable to their issues through the problem solving cases by the other members. Finally, in relation to the theme of the day, the participants set their own objectives and wrote down on the objective card what they would like to achieve for the next week. Table 1 shows the content of the program by session.

**Table 1:** Weekly program of this study

***Week***	***Contents of intervention***	***Specific strategy***
Week 1	Provide information on empowerment. Encourage to introduce themselves and share their experience about living in the nursing home.	
Week 2	Education on communicating skills. Practice communicating skills with peers. Set up their goals for effective communication.	
Week 3	Share successful experience in their lives. Share the ideas for successful nursing home life. Set up their goals for successful nursing home life.	
Week 4–5	Education on effective ways to compliment others Practice to compliment their peers Set up their goals for praising others.	Providing knowledge and skills Active participation, social support Sense of control Self-efficacy
Week 6	Education on steps for problem solving Share ideas for solving the problems related to living in nursing home. Set up their goals for problem solving	
Week 7–8	Education on health issues (physical activity, oral health, and sound sleep). Share their issues on their health issues. Set up their goals for health issues	
Week 9	Share their experience and ideas for what They can do for the nursing home Set up their goals for a successful adjustment to nursing home life.	
Week 10	Review what they have learned. Encourage to announce their supportive friends. Identify whether the goal of the very first session was achieved. Provide a reward for active participation Set up their goals for a happy and satisfying life in the nursing home	

### Measures

Questionnaire was designed to measure general characteristics of study participants, self-esteem, depression, relationship, life satisfaction, and adaptation to facility. General characteristics consisted of gender, age, education, religion, marital status, perceived health, MMSE-K scores, length of stay, and care level. It consisted of 9 items.

Self-esteem scale was developed by Rosenberg (24), and this scale was revised by Ha and Lee (25) for older adults living in nursing homes. The scale was used to measure the self-esteem of the participants. It consists of a total of 10 questions using a 4 points Likert scale. The possible score range was 10–40, and the higher the score of the respondent was, the higher the level of self-esteem. The reliability of this instrument was Cronbach’s α=0.89.

Depression scale was developed by Sheikh and Yesavage (26), and this scale was revised by Cho et al (27). For Korean Geriatric Depression Scale (KGDS). The scale was used to measure the level of depression of the participants. It consisted of a total of 15 questions using Yes (zero score) or No (one score). The possible score range was 0–15, and the higher the score of respondent was, the higher the level of his or her depression. Below 5 scores means no depression, 5 to 9 scores mean possible depression and 10 scores over means severe depression. The reliability of this instrument was Cronbach’s α=0.88.

Relationship Change Scale (RCS) was developed by Schlein and Guerney (28), and this scale was revised by Moon (29) for older adults living in nursing homes. The scale was used to measure the relationship of the participants. It consists of a total of 25 questions using a 5 points Likert scale. The possible score range was 25–125, and the higher the score of the respondent was, the higher the level of relationship. The reliability of this instrument was Cronbach’s α=0.89.

Life satisfaction scale for Korean older adults developed by Yun (30) was used to measure the level of life satisfaction of the participants. It consisted of a total of 20 questions using a 4 points Likert scale. The possible score range was 20–80, and the higher the score of respondent was, the higher the level of his or her life satisfaction. The reliability of this instrument was Cronbach’s α=0.87.

Facility adaptation scale developed by Lee (31) was used to measure the level of facility adaptation of the participants. It consisted of a total of 23 questions using a 5 points Likert scale. The possible score range was 23–115, and the higher the score of respondent was, the higher the level of his or her facility adaptation. The reliability of this instrument was Cronbach’s α=0.91.

### Procedures

This study was approved by the Institutional Review Board of a university in Seoul, South Korea. The researcher visited the nursing home for older adults to obtain the permission for this study. After obtaining the permission from nursing home, researcher contacted the prospective older adult participants living in nursing home, and explained the purpose and objective of the research to them as well as the participation details and the instruments that were to be used in the study. After receiving written consent from willing volunteers, the study samples were selected. Facility adaptation promotion program as an experimental intervention was applied to experimental group by the researcher. In light of the ethical issues, the blood pressure of the members in the control group was measured after the 10-week experiment sessions and post-experiment survey, followed by a training session on health management for 1 hour and 20 minutes. Study variables were measured subsequently before the experiment and at week 10 in both groups. The questionnaire was given only to the older adults who agreed to participate in the study, after which the completed questionnaires were collected. The questionnaires were self-reporting administered by researcher. Each of the participants took approximately 20–25 min to finish the survey. They were informed about the anonymity and the confidentiality of the data they would provide. The data collection period for this study was from August to December 2016.

### Statistics

Data were analyzed using the SPSS version 21.0 (Chicago, IL, USA). Descriptive statistics, the Chi-squared test, and independent *t*-test were used to analyze general characteristics of participants. Homogeneity tests of general characteristics of participants, self-esteem, depression, relationship, life satisfaction, and facility adaptation were analyzed using the Chi-squared test and independent t-test. In order to examine the effects of facility adaptation promotion program, the independent t-test was used. A *P*-value of less than 0.05 was considered statistically significant.

## Results

### General characteristics of study participants

General characteristics of study participants are shown in Table 2. The most of participants was women (experimental: 61.1%, control: 64.9%). Age in the experimental group was average 72.06 years and average 73.02 years in the control group. For the academic background, illiteracy was the most as 41.7% in experimental group, and 46.0% in control group. Most of the participants were alone like widowed or divorced or unmarried (experimental: 83.3%, control: 78.4%), and poor perceived health status (experimental: 50.0%, control: 54.1%). From 6 to 12 month in length of stay was 38.9% in experimental group and 43.3% in control group. As for the general characteristics of the experimental and control groups, as well as the study variables before the experiment, there were no group differences at baseline at a statistical significance level of *P*<0.05 (Table 2).

**Table 2:** General characteristics of the study participants (*N*=73)

***Characteristics***		***Experimental Group (n=36) n(%)***	***Control Group (n=37) n(%)***	χ^2^/t	**P**
Gender	Female	22(61.1)	24(64.9)	0.110	0.811
	Male	14(38.9)	13(35.1)		
Age (yr)	65–74	18(50.0)	20(54.1)		
	75–84	10(27.8)	9(24.3)	0.144	0.930
	≥85	8(22.2)	8(21.6)		
	Mean±SD	72.06*±*5.16	73.02*±*4.96	–2.235	0.532
Education	Illiteracy	15(41.7)	17(46.0)	0.346	0.841
	Elementary school	12(33.3)	10(27.0)		
	Middle school above	9(25.0)	10(27.0)		
Religion	Yes	28(77.8)	30(81.1)	0.122	0.778
	No	8(22.2)	7(18.9)		
Marital status	Married	6(16.7)	8(21.6)	0.289	0.768
	Other†	30(83.3)	29(78.4)		
Perceived health	Healthy	6(16.7)	7(18.9)	0.350	0.839
	Moderate	12(33.3)	10(27.0)		
	Poor	18(50.0)	20(54.1)		
MMSE-K scores	Mean±SD	24.26*±*2.98	24.52*±*3.04	1.267	0.864
Length of stay (month)	3	10(27.8)	10(27.0)	0.163	0.922
	3–6	12(33.3)	11(29.7)		
	6–12	14(38.9)	16(43.3)		
Care level	1	11(30.6)	12(32.4)	0.110	0.947
	2	12(33.3)	13(35.2)		
	3	13(36.1)	12(32.4)		

MMSE-K scores=Mini-mental state examination-K scores.

† Widowed/Divorced/Unmarried.

### Effects of facility adaptation promotion program

Effects of facility adaptation promotion program are presented in Table 3. It was confirmed that the facility adaptation promotion program had statistically significant positive effects on the self-esteem (*t*=19.067, *P*<0.001), depression (*t*=14.491, *P*<0.001), relationship (*t*=24.533, *P*<0.001), life satisfaction (*t*=16.501, *P*<0.001), and adaptation to facility (*t*=24.328, *P*<0.001) for the older adults living in nursing home (Table 3).

**Table 3:** Effects of facility adaptation promotion program (*N*=73)

***Study variables***	***Group***	***n***	***Pre Mean±SD***	***Post***			**t**	**P**
				**t**	P*	***Mean±SD***		
Self esteem	Exp	36	17.42*±*1.94	0.203	0.840	33.53*±*2.97	19.067	<0.001
	Con	37	17.32*±*1.94	17.24*±*3.28				
Depression	Exp	36	10.03*±*1.96	1.051	0.297	4.92*±*2.14	14.491	<0.001
	Con	37	9.51*±*2.21	10.43*±*1.85				
Relationship	Exp	36	50.83*±*8.56	–0.766	0.446	95.53*±*9.98	24.533	<0.001
	Con	37	52.29*±*7.74	46.19*±*5.24				
Life satisfaction	Exp	36	39.58*±*5.60	–0.452	0.653	65.89*±*6.81	16.501	<0.001
	Con	37	40.16*±*5.34	37.19*±*5.06				
Adaptation to facility	Exp	36	44.89*±*6.79	0.641	0.523	89.56*±*8.01	24.328	<0.001
	Con	37	43.92*±*6.13	39.51*±*2.84				

Exp = Experimental group

Con = Control group

## Discussion

The study results showed that after the facility adaptability promotion program, the self-esteem of the experimental group improved in a statistically significant manner, as compared to that of the control group. Such results were identical to those of Chang and Park (10), who used the same program for the elderly in the nursing facilities. These results are also similar to the reports that a 10-week long intervention program offered to multicultural couples for providing emotional support and couple education (23) has significantly increased the self-image and self-esteem of the participants. It is believed that this is because through this intervention program, the elderly in the nursing facilities can discover their strengths and resources, and express positive expectations toward their future to verify their positive self-image. The enhancement of self-esteem of the elderly in a nursing facility would require them to identify their problems and the capacity to take care of themselves through social interaction (7, 10). In order to identify their problems by communicating with people who are under similar conditions and strengthen their problem-solving skills, it would be appropriate to develop intervention strategies for enhancing the self-esteem of the elderly in the facilities that cannot secure their value of existence through interaction among their family members, siblings, or friends. The depression score of the experimental group was significantly lower than that of the control group. This was a similar result to that of Altintas et al (4), who reported that leisure activities had a positive effect on mental activities, depression, facility adaptability, and satisfaction level of the elderly at the facilities, and to that of Konnert et al (14), whose study on cognitive behavior therapy reduced the depression of the elderly at the facilities. Based on the process of identifying and solving problems through dialogues, the elderly at the facilities offered support for each other, the process of which helped lower their depression. Such a result is similar to the positive effect that the patients can get from various self-help groups. After the intervention, the interpersonal score of the experimental group increased significantly, as compared to that of the control group. This result is identical to that of Chang and Park (10), who identified the effect of intervention program from interpersonal aspects. This is also similar to the results of a 10-week intervention program, which improved the interpersonal relationships of the mental patients (22). It is believed that the reason for this is because the developed program included a method that offered information on 'self-expression' and 'effective communication', and encouraged the participants to select trustworthy friends and maintain their friendship. It also offered opportunities to meet people who are in similar situations through group discussions. The program created a platform to develop new relationships that could serve as a substitute for the severed social relationship due to the admission of the elderly to a facility.

The life satisfaction score of the experimental group was found to be significantly higher than that of the control group. This was similar to the results of Altintas et al. (4), which showed that the leisure activities of the elderly at the facilities had a positive effect on their life satisfaction. It was also similar to the results of Chang and Park (10), which showed that an empowerment program for facility adaptation improved the status of the elderly at the facilities and it had a positive effect on their life satisfaction. The life satisfaction of the elderly at the facilities may decrease due to the unfamiliar environment after their admission to the facility (8, 11). However, it is believed that the proposed program would help them develop a sense of intimacy with one another, thereby resulting in an increased level of life satisfaction of the elderly.

The facility adaptation promotion program offered by this study significantly increased the elderly's facility adaptability score, which is similar to the results of a 6-week intervention program that increased the AIDS-infected women's adaptability to a motherly role and the countermeasures (20), as well as those of a previous study, which showed a positive effect on the school adaptability and depression of minority youths, who were vulnerable to committing suicide (21), and an empowerment program that had a positive effect on the elderly's facility adaptability (10). Furthermore, these are identical to others (6, 10), who argued that the adaptability promotion intervention programs would need to be implemented for the improvement of the quality of life of the elderly at the facilities. Considering the reports that showed most of the problems experienced by the elderly are related to environmental or interpersonal changes (10, 32–34), it is believed that the proposed program helped the elderly at the facilities in solving their problems of adaptability by encouraging them to interact with their fellow residents and acquire relevant information. Based on these study results, various efforts by the nursing facilities are required in order to realize the strategies used by the present study, such as helping the elderly form self-help groups to continue meeting with their fellow residents, whom they were introduced to after having been admitted to the facility, and providing them with different opportunities to meet and discuss with others, and to find trustworthy friends in the facility. Furthermore, policy efforts and changes are required in order to regularly apply the adaptability promotion programs to the facility programs and to reinforce the nursing professionals, who have been sufficiently trained on the characteristics of elderly care and group dynamics, and have led such promotion programs, within the nursing facilities.

In study limitations, it is possible that besides the intervention program offered to the experimental group, the researcher's informational and emotional support may have affected the promotion of the experimental group's self-esteem, interpersonal relationship, and facility adaptability. Moreover, the study could not verify the detailed health characteristics of the participants, such as any disease diagnosis, existence of pain, or any medication, and limitations to the elderly in some local facilities, thereby making the study results inadequate to be generalized.

## Conclusion

The effects of facility adaptation promotion program in improving self-esteem, relationship, life satisfaction, and adaptation to facility and decreasing depression were confirmed. Thus, facility adaptation promotion program can be utilized for older adults living in nursing home. Further repetitive studies with larger sample are necessary to confirm the effects more exactly in older adults living in nursing home. At the same time, in-depth qualitative studies are required to understand and analyze the inner world related to adaptation of the older adults living in nursing home.

## Ethical considerations

Ethical issues (Including plagiarism, informed consent, misconduct, data fabrication and/or falsification, double publication and/or submission, redundancy, etc.) have been completely observed by the authors.
